# Spin-flip-driven reversal of the angle-dependent magnetic torque in layered antiferromagnetic Ca_0.9_Sr_0.1_Co_2_As_2_

**DOI:** 10.1038/s41598-022-17206-y

**Published:** 2022-07-27

**Authors:** Jong Hyuk Kim, Mi Kyung Kim, Ki Won Jeong, Hyun Jun Shin, Jae Min Hong, Jin Seok Kim, Kyungsun Moon, Nara Lee, Young Jai Choi

**Affiliations:** Department of Physics, Yonsei University, Seoul, 03722 Korea

**Keywords:** Magnetic properties and materials, Spintronics

## Abstract

Spin-flip transition can occur in antiferromagnets with strong magnetocrystalline anisotropy, inducing a significant modification of the anisotropic magnetic properties through phase conversion. In contrast to ferromagnets, antiferromagnets have not been thoroughly examined in terms of their anisotropic characteristics. We investigated the magnetic-field and angle-dependent magnetic properties of Ising-type antiferromagnetic Ca_0.9_Sr_0.1_Co_2_As_2_ using magnetic torque measurements. An A-type antiferromagnetic order emerges below *T*_N_ = 97 K aligned along the magnetically easy *c*-axis. The reversal of the angle-dependent torque across the spin-flip transition was observed, revealing the strong influence of the magnetocrystalline anisotropy on the magnetic properties. Based on the easy-axis anisotropic spin model, we theoretically generated torque data and identified specific spin configurations associated with the magnetic torque variation in the presence of a rotating magnetic field. Our results enrich fundamental and applied research on diverse antiferromagnetic compounds by shedding new light on the distinct magnetic features of the Ising-type antiferromagnet.

## Introduction

Magnetic anisotropy determines the orientation of spin textures and influences the development of magnetic properties in the presence of a magnetic field^1–4^. The intrinsic magnetocrystalline anisotropy arises from the anisotropy of spin–orbit interaction, which varies with the structure and symmetry of magnetic materials^5–7^. In a permanent ferromagnet, a strong magnetic anisotropy renders high magnetic coercivity and improved maximum energy product^8–11^. A soft ferromagnet exhibits a possibly weak magnetic anisotropy to gain susceptible variation in the net magnetic moment with high permeability^12–15^. Contrarily, the zero net moment that is inherent in antiferromagnets due to staggered spins has often limited the access to their magnetic anisotropy^16,17^. The recent development in antiferromagnetic spintronics, where the antiferromagnetic (AFM) order governs the dynamic transport in single-phase materials, is significant for exploring the magnetic anisotropy of antiferromagnets^18–20^. In this work, the magnetic anisotropy in an Ising-type antiferromagnet has been examined by analyzing the spin-flip-driven reversal behavior of angle-dependent magnetic torques.

Ca_1−*x*_Sr_*x*_Co_2_As_2_ forms a body-centered tetragonal structure, which belongs to the ThCr_2_Si_2_-type structure family^21,22^. In these types of layered compounds, two- or three-dimensional characteristics are possibly formed relying on the interlayer bonding and thus lead to various electronic and magnetic states^23–27^. In Ca_1−*x*_Sr_*x*_Co_2_As_2_ compounds, the magnetic properties can be susceptibly controlled by chemical doping, accompanied by changes in the interlayer magnetic couplings and magnetocrystalline anisotropy via changing the distance between the magnetic layers^21,22^. Without Sr-doping (*x* = 0), an A-type AFM order emerges at *T*_N_ = 74 K, along with the occurrence of a spin–flop transition at *H*_flop_ = 3.7 T for *T* = 10 K. In a 10% Sr-doped compound (*x* = 0.1), the stronger magnetocrystalline anisotropy induces a spin-flip transition with an enhanced Néel temperature, *T*_N_ = 97 K. Further Sr-doping (*x* = 0.2) leads to a complete phase change, resulting in a ferromagnetic state with modified interlayer coupling.

The influence of magnetocrystalline anisotropy is well evidenced in flip/flop transitions^28,29^. In an antiferromagnet, a spin–flop is a field-induced reorientation transition with a relatively weak magnetocrystalline anisotropy^30–32^. In the case of a strong magnetocrystalline anisotropy, a spin-flip transition is observed involving a sudden reversal of the magnetic moments oppositely aligned to the field direction, accompanied by a drastic change in the magnetic properties. A controlled anisotropic phenomenon can help better understand the underlying physics and explore broad spintronic applicability^16^. However, compared to ferromagnets, antiferromagnets have not been thoroughly investigated in terms of their anisotropic properties, including their various origins and controllable factors. Here, we extensively investigated the anisotropy of magnetic properties in a layered Ising-type antiferromagnet Ca_0.9_Sr_0.1_Co_2_As_2_ (CSCA) by conducting magnetic torque (*τ*) measurements. As a vital impact of the magnetocrystalline anisotropy, the reversal of the angle-dependent *τ* across the spin-flip transition was observed. Moreover, we quantified the magnetocrystalline anisotropy and specific spin configurations corresponding to the angle-dependent magnetic *τ* properties by establishing an easy-axis anisotropic spin model^30,33,34^. Our findings can help further explore magnetic anisotropy for various AFM types.

## Results and discussion

**Figure 1:**
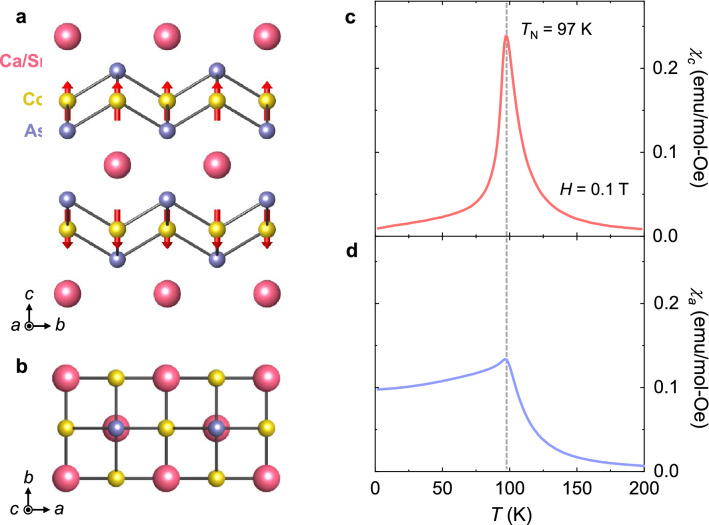
Structure and magnetic susceptibility of antiferromagnetic Ca_0.9_Sr_0.1_Co_2_As_2_ (CSCA). (**a**) Crystallographic structure of body-centered tetragonal CSCA viewed from the *a*-axis. The pink, yellow, and purple spheres represent Ca/Sr, Co, and As atoms, respectively. The red arrow on the Co atom indicates the individual spin direction. The right red arrow indicates the net magnetic moment of each Co_2_As_2_ layer. (**b**) Structure of the CSCA viewed from the *c*-axis. (**c**) Magnetic susceptibility for *H//c* (*χ*_*c*_) as a function of the temperature (*T*), taken upon warming at *H* = 0.1 T after zero-field cooling. (**d**) Magnetic susceptibility for *H//a* (*χ*_*a*_) as a function of *T* at *H* = 0.1 T. The vertical line denotes the onset of antiferromagnetic (AFM) order, *T*_N_ = 97 K.

The CSCA crystallizes in a tetragonal structure (*I4/mmm* space group) with lattice constants *a* = 0.408 nm and *c* = 1.086 nm. As shown in the crystallographic structures (Fig. 1a and b), two magnetic Co_2_As_2_ layers are located opposite each other around the center of a unit cell and are separated by a nonmagnetic Ca/Sr layer. Ising-type Co spins, which are ferromagnetically ordered within a layer, are correlated antiferromagnetically to the spins in the neighboring layer^21,22^. This leads to an A-type AFM order along the *c*-axis, which emerges at *T*_N_ = 97 K. The magnetic susceptibility, defined as the magnetization (*M*) divided by the magnetic field (*H*), *χ* = *M/H*, measured at *H* = 0.1 T on warming after zero-*H*-cooling, was measured for *H//c* (*χ*_*c*_) and *H//a* (*χ*_*a*_), as shown in Fig. 1c and d, respectively. An anomaly occurs at *T*_N_, and the fast inclination of *χ*_*c*_ below *T*_N_ presents spins mainly aligned along the *c*-axis, consistent with the Ising-type AFM order.

**Figure 2:**
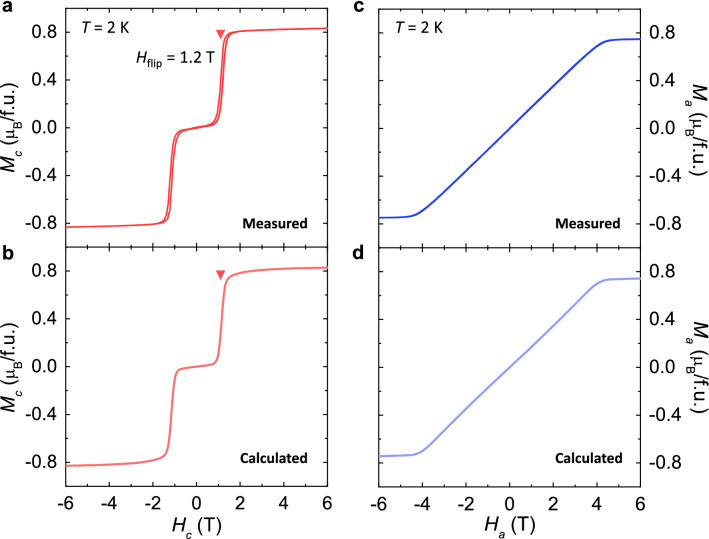
Measured and calculated isothermal magnetizations. (**a**) *H*-dependence of magnetization (*M*) for *H//c* (*M*_c_), measured at *T* = 2 K. The red inverted triangle denotes the occurrence of the spin-flip transition, *H*_flip_ = 1.2 T. (**b**) *H*-dependence of *M*_c_, calculated from the easy-axis anisotropic model. Parameters obtained by fitting are given by $g{\mu }_{B}{H}_{{\rm flip}}/JS=2$, where $S$ is the saturation magnetic moment, and $K=1.4 J{S}^{2}$. (**c**) *H*-dependence of magnetization (*M*) for *H//a* (*M*_a_), measured at *T* = 2 K. (**d**) Calculated *H*-dependence of *M*_a_.

A two-sublattice model in which two *M* vectors with equal magnitudes are oriented oppositely in a zero *H*-field is typically adopted to describe a collinear AFM type^35^. Under a sufficient magnitude of *H* along the AFM easy axis, a magnetic phase transition would occur, resulting in flops or flips of the *M* vectors^36,37^. This spin reorientation phenomenon through phase conversion is signified by distinct anomalies in the magnetic properties. In our CSCA, a spin-flip transition was observed as a substantial step-like increase in *M*_*c*_ (*M* along the *c*-axis) at *H*_flip_ = 1.2 T and *T* = 2 K (Fig. 2a). In addition, a first-order nature of the magnetic transition is signified by a magnetically hysteretic behavior across *H*_flip_. After *H*_flip_, a slight linear slope was found, ascribed to the thermal fluctuation precluding the saturation of *M*_*c*_. At *H* along the magnetically hard *a*-axis, *M*_*a*_ (*M* along the *a*-axis) exhibits a linear inclination, reflecting the gradual canting of the Co spins (Fig. 2c). Above ~ 4 T, the slope of *M*_*a*_ is significantly reduced.

The essential feature of the spin-flip transition was theoretically investigated by hosting an easy-axis anisotropic spin model^38^. The model Hamiltonian is composed of exchange interaction, Zeeman energy, and magnetocrystalline anisotropy (see Method in detail). The magnetic energies for the AFM and spin-flip phases are given as ${E}_{{\rm AF}}=-2J{S}^{2}$ and ${E}_{{\rm flip}}=2J{S}^{2}-2g{\mu }_{{\rm B}}HS$, respectively, where $J$ represents the AFM coupling strength, $g$ = 2, and $S$ represents the net moment of the Co_2_As_2_ layers. For a large magnetocrystalline anisotropy constant, $K$, the condition of ${E}_{{\rm AF}}= {E}_{{\rm flip}}$ at *H*_flip_ gives rise to $g{\mu }_{B}{H}_{{\rm flip}}/JS=2$. $K=1.4 J{S}^{2}$ can also be estimated by matching the theoretical results of *M*_*c*_ and *M*_*a*_ (Fig. 2b and d) with the experimental data at 2 K (Fig. 2a and c), which corresponds to the strong magnetocrystalline anisotropy regime, as $K>J{S}^{2}$ for our spin model. In the experimental data, the AFM phase transforms gradually to the spin-flip phase with a certain broadness and magnetic hysteresis of the transition. This can be attributed to the phase coexistence between the AFM and spin-flip phases. The spatial modulations regarding the first-order characteristic of the *H*-induced transition were inherently incorporated into the calculations by considering the scale of a spin cluster within a layer (see Methods and Supplementary Information S1). The resulting *M*_*c*_ and *M*_*a*_ are presented in Fig. 2b and d, reproducing the anisotropic magnetic properties observed experimentally.

**Figure 3:**
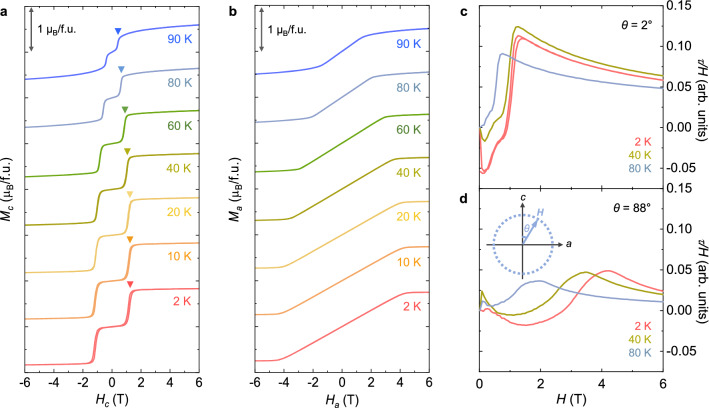
Temperature evolution of anisotropic magnetizations and anisotropic magnetic torques. (**a**) Isothermal *M*_*c*_ measured at various temperatures, *T* = 2, 10, 20, 40, 60, 80, and 90 K. The *M*_*c*_ data are shifted vertically for clear visualization. A marked scale indicates 1 μ_B_/f.u. Inverted triangles represent the occurrence of *H*_flip_ at each *T*. (**b**) Isothermal *M*_*a*_ measured at various temperatures, *T* = 2, 10, 20, 40, 60, 80, and 90 K. (**c**) Magnetic torque divided by the magnetic field, *τ/H*, measured at *θ* = 2° for *T* = 2, 40, and 80 K. (**d**) Magnetic torque divided by the magnetic field, *τ/H*, measured at *θ* = 88° for *T* = 2, 40, and 80 K. Inset shows the geometry of applied *H* in the *ac* plane. *θ* = 0° for the *c*-axis and *θ* = 90° for the *a*-axis.

In our CSCA, the magnetocrystalline anisotropy tends to bind the spin directions to the *c*-axis, leading to a stable AFM phase in zero *H*. *H* along the *c*-axis (*H*_*c*_) triggers spin flips, exhibiting a significant increase in *M*_c_. As shown in the *T* evolution of *M*_c_ (Fig. 3a), *H*_flip_ for *M*_*c*_ is gradually lowered, and the step-like feature is continually reduced upon increasing *T*. *H*_flip_ at 90 K is reduced by approximately one-third of that at 2 K. Contrarily, the slight slope after conversion to the flipped state at 2 K is progressively increased with increasing *T*. This trend reflects the inclusion of thermal fluctuations, which interferes with the full saturation of *M*_c_. Owing to high magnetic anisotropy, *H*_*a*_ generates steadily canted net moments (Fig. 3b). *H*_*a*_, above which the slope is significantly declined, moves to a lower *H* upon increasing *T*.

The anisotropic magnetic properties and occurrence of spin-flips were also examined by measuring the magnetic torque per unit volume, ***τ*** = ***M*** × ***H***, as shown in Fig. 3c and d. *H* is rotated in the *ac* plane with an angle *θ* deviating from the *c*-axis as depicted in the schematic (inset of Fig. 3d) (*θ* = 0° for the *c*-axis and *θ* = 90° for the *a-*axis). The value of *τ* is zero at certain angles because of the parallel or antiparallel alignment between *M* sublattices and *H* at *θ* = 0 and 180°; further, there is a cancellation of *τ* with equal magnitude and opposite sign for two *M* sublattices at *θ* = 90 and 270°. Close to *θ* = 0 and 90°, we observed strongly anisotropic signals of *τ/H*. At *θ* = 2°, a precipitous increase in *τ/H* was found with magnetically hysteretic behavior at *H*_flip_ = 1.2 T and *T* = 2 K (Fig. 3c). At *T* = 40 and 80 K, *H*_flip_ is continuously reduced, in accordance with the measured *M*_c_ in Fig. 3a. At *θ* = 88°, the broad variation by sweeping *H* for *T* = 2, 40, and 80 K indicates the largely anisotropic nature of CSCA crystals (Fig. 3d).

**Figure 4:**
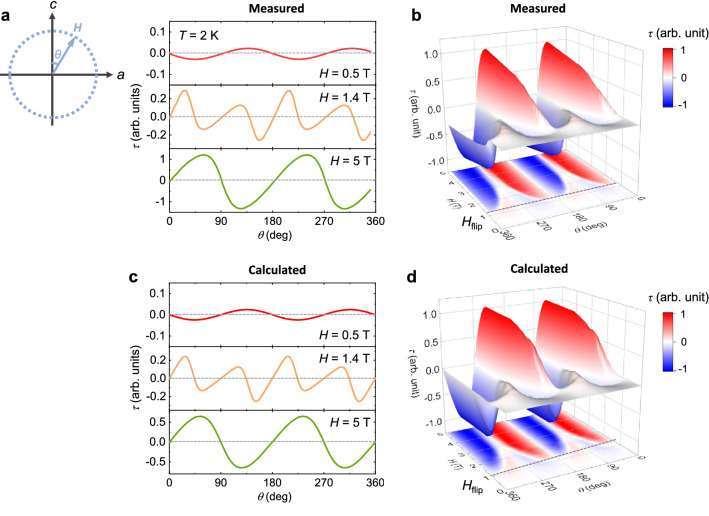
Measured and calculated magnetic torques at 2 K. (**a**) Angle dependence of the magnetic torque per unit volume,* τ* = ***M*** × ***H***, measured at *T* = 2 K by rotating *H* in the *ac* plane at *H* = 0.5, 1.4, and 5 T. As shown in a schematic, *θ* is deviated from the *c*-axis. The scale of *τ* at 5 T is ten times greater than that at 0.5 T. (**b**) 2D and 3D contour plots obtained from the angle-dependent *τ* data measured at various values of *H* and *T* = 2 K. The dotted line denotes the occurrence of a spin-flip transition, *H*_flip_ = 1.2 T. (**c**) Calculated angle dependence of the magnetic *τ* at *H* = 0.5, 1.4, and 5 T. (**d**) 2D and 3D contour plots established from the calculated angle-dependent *τ* data.

A detailed evolution of the anisotropic *M* through the spin-flips is identified by the angular dependence of magnetic *τ*, shown in Fig. 4. Under a low *H*, that is, *H* = 0.5 T, a small sinusoidal variation in *τ* can be observed (Fig. 4a). In the spin-flip regime (*H* = 1.4 T), *τ* values near *θ* = 0 and 180° are partially reversed, which could be attributed to the adjusted anisotropic properties from the enhanced *M*_*c*_ through the spin-flip transition. However, the positive slope of *τ* near *θ* = 90 and 270° is maintained. *τ* is completely reversed with a significantly enhanced magnitude of *τ* at *H* = 5 T, considerably higher than *H*_flip_ because the small rotation of *H* near *θ* = 90 and 270° generates a sufficient *c*-axis component that triggers the flip transition. In the *H*–*θ* contour plot (Fig. 4b), two features can be clearly seen across the spin-flip transition: an *H-*driven reversal of *τ* and a much greater *τ* variation above *H*_flip_. The angular dependence of magnetic *τ* has been estimated, as shown in Fig. 4c. The calculated *τ* data at *H* = 0.5 T reveal the same sinusoidal variation as the measured *τ*. Across *H*_flip_, partial and entire reversals of *τ* at *H* = 1.4 and 5 T could also be presented. The results suggest that the easy-axis anisotropic spin model with strong magnetocrystalline anisotropy could serve as an efficient tool to verify the variation in the anisotropic magnetic properties in Ising-type antiferromagnets. In Fig. 4d, the estimated *H*–*θ* contour plot agrees well with the experimental result.

**Figure 5:**
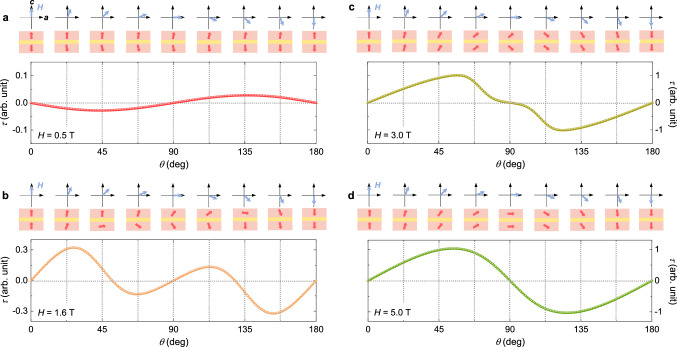
Detailed spin configurations for angle-dependent torques. (**a**) Angle dependence of magnetic *τ*, calculated by fitting to the experimental *τ* data at *T* = 2 K. *H* is rotated in the *ac* plane at *H* = 0.5 T. The schematics designate the configurations of the net magnetic moments with respect to the *H* directions. (**b–d**) Calculated angle-dependent *τ* and corresponding net-moment arrangements at *H* = 1.6, 3, and 5 T, respectively.

Moreover, the easy-axis spin model can help identify definitive spin configurations during the rotation of *H*, clarifying the close relationship between the various spin states and magnetic *τ* data. With the rotation of a weak *H* = 0.5 T from 0 to 45°, the net moments of the two layers are only slightly canted because of the strong magnetocrystalline anisotropy (Fig. 5a). The *H* direction adjacent to the net moment in the upper layer generates a small negative *τ* value. At *H* with *θ* = 90°, the equal and tiny canting angles of the net moments toward the *a*-axis lead to zero *τ*. With further rotation of *H* to *θ* = 135°, the *τ* value changes to positive with the *H* direction close to the net moment in the lower layer. In this manner, *τ* exhibits a small magnitude but an evidently sinusoidal angle variation. At *H* = 1.6 T, just after the flip transition, the partially reversed behavior of *τ* near *θ* = 0 and 180° can be explained by the detailed configurations of the net moments shown in Fig. 5b. The net moments that are parallel at *θ* = 0° convert to the canted arrangement in which the net moment in the lower layer is located closer to the *H* direction, resulting in a positive *τ* value at *θ* = 22.5°. Further rotation of *H* to *θ* = 67.5° transforms the *τ* state from positive to negative by orienting the net moment in the upper layer closer to *H*. Similar sign variations are repeated by fully rotating *H*. At *H* = 3 T, the angle-dependent *τ* is mostly reversed, as plotted in Fig. 5c. For *θ* up to 67.5°, the net moments in both the layers move together but are rotated less than *H* under the influence of strong magnetocrystalline anisotropy. This leads to a positive *τ* state. Above *θ* = 67.5°, the angles of the two net moments begin to split and become equal at *θ* = 90°, with *H* being insufficient to saturate the net moments along the *a*-axis. The similar canted angles of the net moments near *θ* = 90° result in a plateau-like behavior. At *θ* over 90°, *H*, which contains a negative *c* component, makes the net moments turn closer to the negative *c*-axis and generates a negative *τ* value. Under a strong *H* = 5 T sufficient to saturate the magnetic moments along both the *a-* and *c*-axis, the overall angle-dependent *τ* is completely reversed with a largely enhanced magnitude of *τ* (Fig. 5d). The net moments in both layers tend to move collectively.

**Figure 6:**
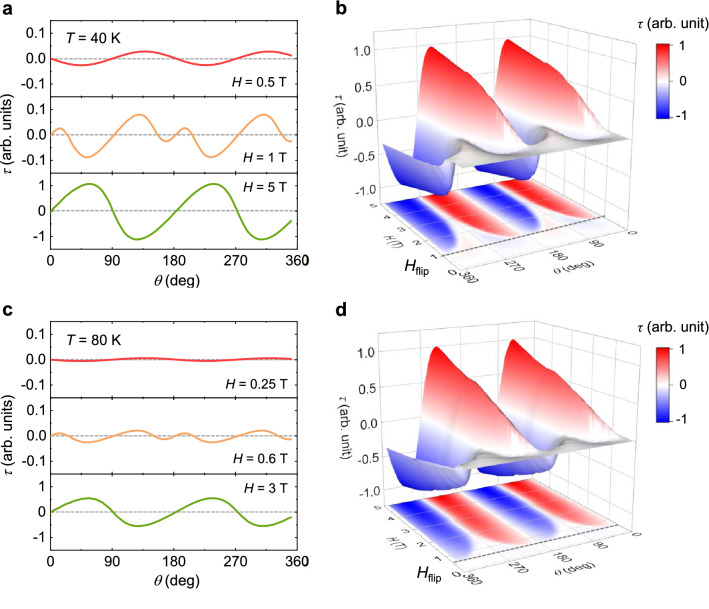
Temperature evolution of angle-dependent magnetic torques. (**a**) Angle dependence of the magnetic torque per unit volume,* τ* = ***M*** × ***H***, measured at *T* = 40 K by rotating *H* in the *ac* plane at *H* = 0.5, 1, and 5 T. (**b**) Contour plots constructed from the angle-dependent *τ* data measured at various values of *H* and *T* = 40 K. The dotted line signifies the occurrence of spin-flip transition, *H*_flip_ = 1.0 T (**c**) Angle dependence of *τ*, measured at *T* = 80 K by rotating *H* in the *ac* plane at *H* = 0.25, 0.6, and 3 T. (**d**) Contour plot for *T* = 80 K. The dotted line denotes the occurrence of *H*_flip_ = 0.6 T.

As *T* is increased, similar angle dependences with reduced overall *τ* values could be observed, as shown in the *τ* data acquired at *T* = 40 and 80 K in Fig. 6. The *H*-driven reversal of *τ* remains intact at *T* = 40 and 80 K. At 40 K, the rotation of *H* = 0.5 T in the *ac* plane leads to small but sinusoidal variations in *τ*, similar to that measured at *T* = 2 K, as shown in Fig. 6a. The spin-flip transition occurs at lower *H*, *H*_flip_ = 1 T, as shown in the *H*–*θ* contour plot (Fig. 6b). At *H*_flip_ = 1 T, the change in the anisotropic properties also induces a partial reversal of the *τ* values near *θ* = 0°. In a strong *H* = 5 T, the overall magnitude of *τ* is largely enhanced with the sign of *τ* entirely reversed, indicating that the *c*-axis component that hosts the spin flips is induced by a slight deviation in *H* from *θ* = 90°. While the overall *τ* values are significantly reduced at *T* = 80 K (Fig. 6c), the evolving behavior by changing the anisotropy of the magnetic properties via the flip transition remains in comparison with the spin flips arising at *H*_flip_ = 0.6 T, shown in the *H*–*θ* contour plot (Fig. 6d).

## Conclusions

In summary, we explored the anisotropic magnetic properties of a layered Ising-type antiferromagnet, Ca_0.9_Sr_0.1_Co_2_As_2_, using a magnetic torque measurement. Interlayer antiferromagnetic couplings lead to alternatingly arranged net magnetic moments along the easy *c*-axis, forming an A-type antiferromagnetic order below *T*_N_ = 97 K. Under the magnetic field along the *c*-axis, a spin-flip transition occurs at *H*_flip_ = 1.2 T and *T* = 2 K, and it gradually decreases with increasing temperature. As a consequence of the strong magnetocrystalline anisotropy, we demonstrated the progressive reversal of the angular-dependent magnetic torques through the spin-flip transition. Additionally, by adopting an easy-axis spin Hamiltonian, we could estimate the diverse spin states formed by rotating magnetic fields, with the calculated magnetic torque data well-matching the experimental results. The scheme used in this work can be extended to diverse antiferromagnetic compounds.

## Methods

### Single-crystal growth

We synthesized Ca_0.9_Sr_0.1_Co_2_As_2_ single crystals using the self-flux method^21^. A precursor of CoAs was prepared in advance by the solid-state reaction using mixed powders of Co and As with a molar ratio of 1:1, followed by calcination at 700 °C for 24 h in a furnace. The precursor was mixed with Ca and Sr flakes, and the mixture was placed in an alumina crucible, which was vacuum-sealed in a quartz tube. The quartz tube was dwelled at 1280 °C for 16 h in a high-temperature furnace, steadily cooled to 850 °C at a rate of 2 °C/h, and then fast cooled to room temperature at a rate of 100 °C/h. Sizable crystals were attained with typical dimensions of 1.5 × 3 × 0.2 mm^3^.

### Magnetization and magnetic torque measurements

The temperature and magnetic-field dependences of the DC magnetizations were measured using a vibrating sample magnetometer (VSM) at *T* = 2–200 K and *H* =  − 6–6 T in a physical properties measurement system (PPMS, Quantum Design, Inc.). The magnetic torque was measured using a torque magnetometer option in the PPMS equipped with a single-axis rotator. The crystal was mounted on a piezoresistive cantilever of the chip included in the option (P109A, Quantum Design, Inc.). A Wheatstone bridge circuit allows detecting small changes in the torque.

### Spin Hamiltonian for the Ising-type antiferromagnet

The magnetic Hamiltonian per number of Co moments in a single layer, $N$, with a periodic boundary condition for an easy-axis magnetocrystalline anisotropy is given by $\frac{\mathcal{H}}{N}=J\sum_{i=1}^{2}{\overrightarrow{S}}_{i}\cdot {\overrightarrow{S}}_{i+1}- g{\mu }_{B}\overrightarrow{H}\cdot \sum_{i=1}^{2}{\overrightarrow{S}}_{i}+K\sum_{i=1}^{2}{sin}^{2}{\theta }_{i}.$

The first term represents the antiferromagnetic interaction between the Co moments in adjacent layers, where $J$ is the coupling strength. In the second term describing the Zeeman energy, $g$ = 2, and the magnetic field $\overrightarrow{H}$ lies on the *ac* plane, making an angle *θ* with the *c*-axis. The uniaxial magnetocrystalline anisotropy energy is added to the third term, compatible with the favorable spin orientation along the *c*-axis; $K$ denotes the magnetocrystalline anisotropy constant. To consider the three-dimensional nature of our system, the magnetic moment of each ferromagnetic Co_2_As_2_ layer at a given *T* and *H* is described as ${S}_{i}\left(T, H\right)={S}_{i}\left(T,0\right)+a\left({S}_{i}\left({0,0}\right)-{S}_{i}(T,0)\right)H+b{(S}_{i}\left({0,0}\right)-{S}_{i}\left(T,0\right)){H}^{2}$, where the coefficients are $a=$ 1.0 × 10^−3^ T^−1^ and $b=$ 8.9 × 10^−3^ T^−2^ for *T* = 2 K, and $a=$ 4.0 × 10^−2^ T^−1^ and $b=$ 8.9 × 10^−3^ T^−2^ for *T* = 80 K; ${S}_{i}\left({0,0}\right)$ = 0.42 μ_B,_ ${S}_{i}\left(2 {\mathrm{K}},0\right)$ = 0.42 μ_B_, and ${S}_{i}\left(80 {\mathrm{K}},0\right)$ = 0.26 μ_B_. In the effective quasi-one-dimensional system, the spin ${S}_{i}$ within a layer is replaced by ${S}_{i}\left(T, H\right).$ The isothermal magnetization *M*_c_ can be calculated by the following formula: $\langle {M}_{c}(H)\rangle =\frac{1}{Z}{\mathrm{Tr}}[{M}_{c}(H){e}^{-\widetilde{\mathcal{H}}/{k}_{B}{T}_{{\rm eff}}}]$, where $Z$ is the partition function, ${k}_{B}$ is the Boltzmann constant, $\widetilde{\mathcal{H}}\equiv \mathcal{H}/N$, and ${M}_{c}\left(H\right)= \widehat{H}\cdot \sum_{i=1}^{2}{\overrightarrow{S}}_{i}\left(T,H\right)$. The effect temperature is given by ${T}_{eff}=T/{n}_{c}(T)$, where ${n}_{c}(T)$ represents the average number of spins in a ferromagnetic spin cluster within a layer at a finite *T*. When $T$ approaches zero, ${n}_{c}(T)$ approaches infinity since the spins are completely aligned.

## Supplementary Information

Supplementary Information.

## Data Availability

The datasets generated and/or analysed during the current study are available in the Crystallography Open Database (COD) repository, #3000403.
