# Supplementary material for: Spin-flip-driven reversal of the angle-dependent magnetic torque in layered antiferromagnetic Ca0.9Sr0.1Co2As2
Source: Sci Rep. 2022 Jul 27;12:12866. doi: 10.1038/s41598-022-17206-y (PMC9329288; doi:10.1038/s41598-022-17206-y)
Supplement: Supplementary file 1 — Supplementary Information. [file 41598_2022_17206_MOESM1_ESM.pdf]

Supplementary Information for **Spin-flip-driven reversal of the angle-dependent magnetic torque in layered antiferromagnetic $Ca_{0.9}Sr_{0.1}Co_2As_2$**

Jong Hyuk Kim, Mi Kyung Kim, Ki Won Jeong, Hyun Jun Shin, Jae Min Hong, Jin Seok Kim, Kyungsun Moon, Nara Lee*, and Young Jai Choi*

Department of Physics, Yonsei University, Seoul 03722, Korea

These authors contributed equally: J. H. Kim, M. K. Kim.

Correspondence and requests for materials should be addressed to Y. J. C. (phylove@yonsei.ac.kr) or N. L. (eland@yonsei.ac.kr).

## S1. Spin-flop and spin-flip transitions in $CaCo_2As_2$ and $Ca_{0.9}Sr_{0.1}Co_2As_2$ crystals

In the $Ca_{1-x}Sr_xCo_2As_2$ compounds, interlayer magnetic couplings and magnetocrystalline anisotropy seem very sensitive to the interlayer distance, which can be manipulated by chemical doping.[1] To clarify this, we applied our uniaxial spin model to the isothermal magnetization at 4 K for a bare $CaCo_2As_2$ (CCA) crystal, as displayed in Fig. S1(a) and (b). It appears that the exchange interaction energy ($JS^2 = 1.44 \times 10^5$ J/m$^3$) in CCA is larger than that of $Ca_{0.9}Sr_{0.1}Co_2As_2$ (CSCA) ($JS^2 = 5.66 \times 10^4$ J/m$^3$), owing to the robustness of antiferromagnetic order and occurrence of magnetic transition at $H_{flop}$ = 3.7 T in CCA. However, whether the spin-flop or spin-flip transition occurs can be determined using the ratio of magnetocrystalline anisotropy energy ($K$) to $JS^2$. The ratio has been estimated as $K/JS^2 = 0.3$ for CCA. This belongs to a weak magnetocrystalline anisotropy regime that leads to a spin-

flop transition rather than a spin-flip transition, as opposed to the CSCA with $K/JS^2 = 1.4$. In addition, a spin-flop transition is signified by the extrapolation of the linear slope above $H_{\mathrm{flop}}$ that merges at the origin, as shown in Fig. S1(a) for CCA. The slope of magnetization along the $c$-axis ($M_c$) after $H_{\mathrm{flop}} = 3.7$ T indicates the additional canting of flopped state (CCA), which should be larger than the slope of $M_c$ in the flipped state (CSCA). In the previous study, the 20% Sr-doped compound ($x = 0.2$) revealed a complete phase change resulting in a ferromagnetic phase. After further Sr-doping ($x = 0.3$), another antiferromagnetic phase with easy-$ab$-plane was observed.[1] The sign variation of $J$, which is susceptible to the $c$-axis parameter, is plausibly attributed to RKKY-like exchange interactions.

In the first-order magnetic phase transition, the transition does not occur abruptly through a sharp jump of magnetization. As shown in the experimental $M_c$ data (Fig. S1(c) and Fig. 2(a) in the main manuscript), the antiferromagnetic phase changes continually to the spin-flip phase, which results in a certain broadness and magnetic hysteresis of the transition. Therefore, the specific smoothening of the spin-flip transition, including the nonlinear regime after $H_{\mathrm{flip}}$, can be attributed to the phase coexistence between the antiferromagnetic and spin-flip phases formed in the transition regime. The spatial modulations were inherently included in the theoretical calculations by considering the scale of a spin cluster within a layer.

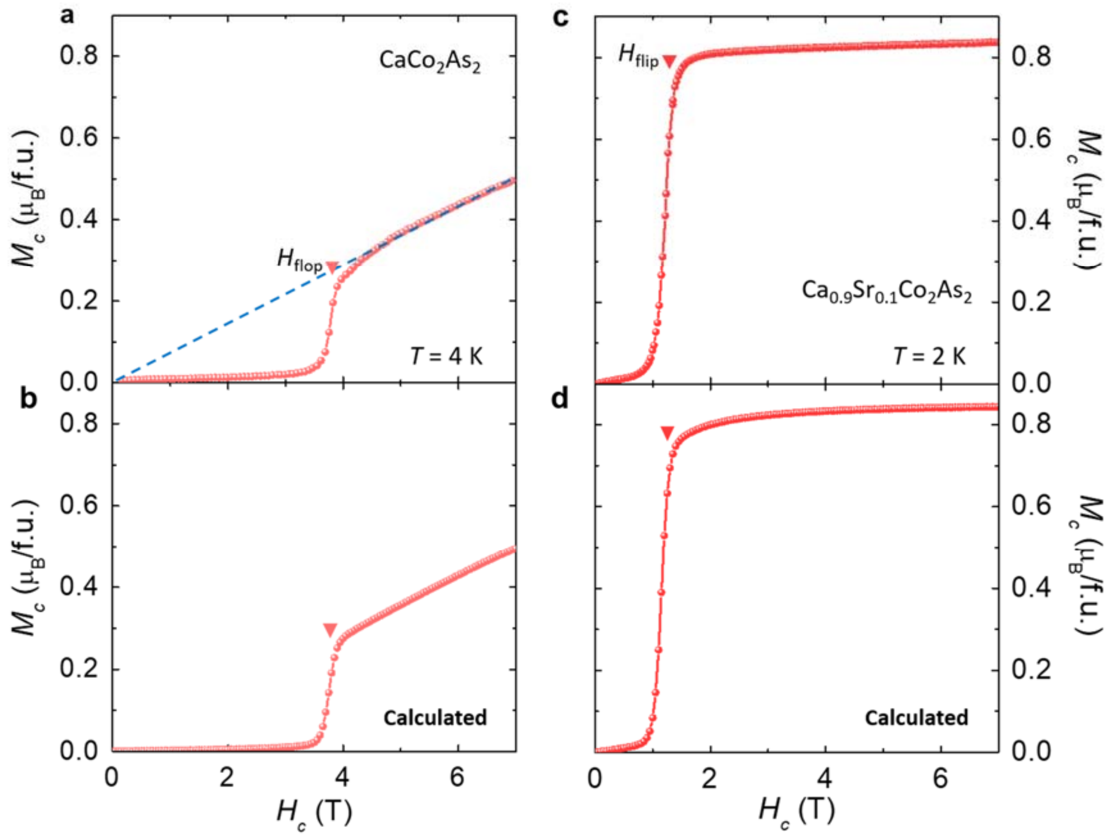


**Fig. S1 Spin-flop and spin-flip transitions in $CaCo_2As_2$ and $Ca_{0.9}Sr_{0.1}Co_2As_2$ crystals.** (a) Isothermal magnetization along the *c* axis, $M_c$, at $T = 4$ K for a CCA crystal. The data were digitalized from the plot in J. J. Ying *et al.*, *Physical Review B* **85**, 214414 (2012). The red inverted triangle denotes the occurrence of the spin-flop transition, $H_{flop} = 3.7$ T. The blue dashed line is the extrapolation of the linear slope above $H_{flop}$ that merges at the origin. (b) Calculated $M_c$ for the CCA. (c) $M_c$ measured at $T = 2$ K for a CSCA crystal. The red inverted triangle designates the occurrence of the spin-flip transition, $H_{flip} = 1.2$ T. (d) Calculated $M_c$ for the CSCA.

## S2. Analysis of isothermal magnetizations at $T = 80$ K

At $T = 80$ K near $T_N$, fitting to the experimental data gives rise to calculated $M_c$ and $M_a$ (Fig. S2) with significantly reduced $JS^2 = 2.33 \times 10^4$ J/m$^3$, but the relative ratio is maintained as $K/JS^2 = 1.4$. These aspects can be understood by considering thermal softening, which causes a substantial reduction of the model parameters $J$ and $K$ as $T$ rises.[2,3] As stated before, the

spin-flip transition occurs under the condition of large magnetocrystalline anisotropy, i.e., $K > JS^2$, and a spin-flop can be identified by the extrapolation of the slope above $H_{flop}$ that merges at the origin. Noticeably, the condition for the occurrence of spin-flip transition, $K/JS^2 = 1.4$, remained intact and the signified slope feature for spin-flops was not observed at $T$ = 80 K for CSCA. While the full saturation of $M_c$ is hindered by thermal fluctuations at finite temperatures, the $M_c$ behavior can be considered within the spin-flip nature.

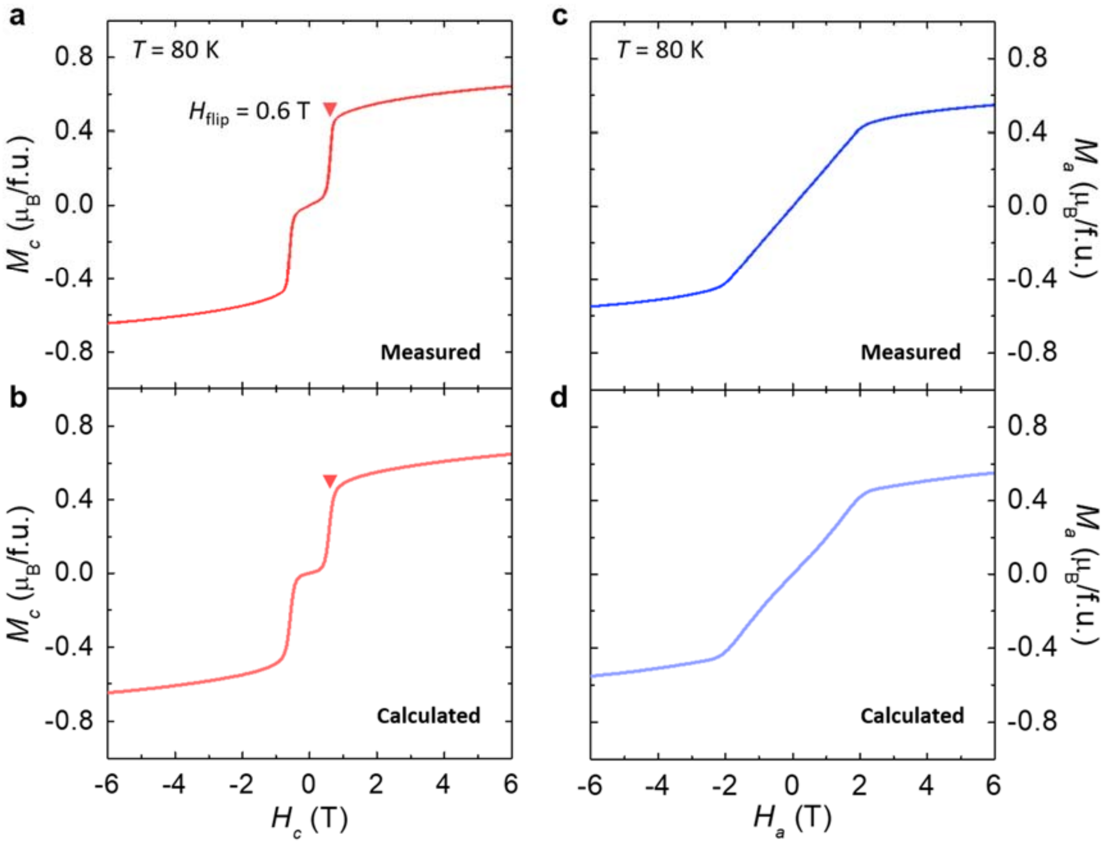


**Fig. S2 Measured and calculated isothermal magnetizations at 80 K.** (a) $M_c$ measured at $T$ = 80 K for CSCA. The red inverted triangle denotes the occurrence of the spin-flip transition, $H_{flip}$ = 0.6 T. (b) $M_c$ theoretically estimated for the data shown in (a). (c) $M_a$ measured at $T$ = 80 K for CSCA. (d) $M_a$ theoretically estimated for the data shown in (c).

## References


1. Ying, J. J. *et al.* The magnetic phase diagram of $Ca_{1-x}Sr_xCo_2As_2$ single crystals. *Europhys. Lett.* **104**, 67005 (2013).
2. Heine, M., Hellman, O. & Broido, D. Temperature-dependent renormalization of magnetic interactions by thermal, magnetic, and lattice disorder from first principles. *Phys. Rev. B* **103**, 184409 (2021).
3. Miura, D. & Sakuma, A. Temperature Dependence of Magnetocrystalline Anisotropy in Itinerant Ferromagnets. *Journal of the Physical Society of Japan* **91**, 023706 (2022).
